# A protective role for autophagy in vitiligo

**DOI:** 10.1038/s41419-021-03592-0

**Published:** 2021-03-25

**Authors:** Emanuela Bastonini, Daniela Kovacs, Salvatore Raffa, Marina delle Macchie, Alessia Pacifico, Paolo Iacovelli, Maria Rosaria Torrisi, Mauro Picardo

**Affiliations:** 1 Cutaneous Physiopathology and Integrated Center of Metabolomics Research, San Gallicano Dermatological Institute, IRCCS, Rome, Italy; 2 Ultrastructural Pathology Lab., Medical Genetics and Advanced Cellular Diagnostics Unit, Sant’Andrea University Hospital & Department of Clinical and Molecular Medicine, Sapienza University of Rome, Rome, Italy; 3 Clinical Dermatology, San Gallicano Dermatological Institute, IRCCS, Rome, Italy

**Keywords:** Macroautophagy, Translational research

## Abstract

A growing number of studies supports the existence of a dynamic interplay between energetic metabolism and autophagy, whose induction represents an adaptive response against several stress conditions. Autophagy is an evolutionarily conserved and a highly orchestrated catabolic recycling process that guarantees cellular homeostasis. To date, the exact role of autophagy in vitiligo pathogenesis is still not clear. Here, we provide the first evidence that autophagy occurs in melanocytes and fibroblasts from non-lesional skin of vitiligo patients, as a result of metabolic surveillance response. More precisely, this study is the first to reveal that induction of autophagy exerts a protective role against the intrinsic metabolic stress and attempts to antagonize degenerative processes in normal appearing vitiligo skin, where melanocytes and fibroblasts are already prone to premature senescence.

## Introduction

Vitiligo, the most common hypopigmentary disorder, is mainly an autoimmune disease, however the events leading to the decline in self-tolerance toward melanocytes are not known. Accumulating evidence argues for some intrinsic metabolic defects in vitiligo melanocytes that may lead to the acquisition of a senescence-associated secretory phenotype^1,2^ and perhaps activate immune cells.

Recently, Qiao and co-workers linked the biological functions and the redox homeostasis of melanocytes to the autophagy process, proving that suppression of ATG7-dependent autophagy inhibits proliferation and results in premature senescence of epidermal melanocytes. Vice versa, overexpression of autophagy protects melanocytes from oxidative stress-induced apoptosis^3^.

To date, research surrounding the role of autophagy in Vitiligo pathogenesis has been focused on melanocyte cell line PIG3V and lesional areas of patients^4,5^. However, the exact role of this process in the disease is still not clear.

Autophagy is an evolutionarily conserved and highly orchestrated catabolic recycling process that guarantees cellular homeostasis by selective removal of aberrant misfolded or long-lived proteins, damaged lipids, and dysfunctional or senescent subcellular organelles that are delivered to the lysosomal compartment where they are degraded^6,7^. However, glucose and/or adenosine triphosphate (ATP) deprivation, oxidative stress and compromised mitochondrial function may result in the accumulation of cytotoxic mediators and energy depletion. Consequently, the autophagic machinery may intervene as a “homeostatic rheostat” to maintain energy balance and nutrient supply. Indeed, its dysregulation would hinder the ability of cells to sustain metabolic needs and might contribute to the onset and development of some metabolic disorders including insulin resistance, diabetes mellitus, obesity, and atherosclerosis^8–10^. Autophagy is regulated by different cell signaling pathways of which two kinases, belonging to intersecting pathways, are key players in the orchestration of its control. Adenosine monophosphate (AMP)-activated protein kinase (AMPK) positively regulates autophagy^11^. This kinase is activated by reduced energy charge, which it senses through increases in the AMP/ATP ratio. In contrast, mammalian target of rapamycin (mTOR), which regulates the intracellular metabolic state by shifting catabolism to growth-promoting anabolism, is a negative suppressor of autophagy^12^. Different metabolic states regulate mTOR. For instance, under depletion or low glucose concentrations, cells undergo a metabolic switch from aerobic glycolysis to respiratory growth and hexokinase II (HKII), the mitochondria-located enzyme that catalyzes the first step of glycolysis, binds to mTORC1 to inhibit its downstream signaling and induce autophagy^13^. Moreover, mTOR kinase activity may be suppressed by the activation of AMPK itself^11^.

A number of different and apparently contradictory biological roles are covered by autophagy. Some papers described autophagy as a programmed cell death mechanism. By contrast, a growing number of studies supports the existence of a dynamic interplay between metabolism and autophagy^14^, whose induction can result more in a survival-prone response with a protective role in counteracting damage caused by several stress conditions^15,16^.

We previously demonstrated that melanocytes obtained from normally pigmented vitiligo skin show cellular and molecular alterations characterized by constitutive activation of antioxidant enzymes^1^ and defects in mitochondrial metabolism reflected in altered expression and activity of complex I, increased generation of reactive oxygen species (ROS), low ATP production, and modified expression of some glycolytic enzymes^2^. In line with these data, here we provide, to the best of our knowledge, the first description of autophagy in melanocytes and fibroblasts from non-lesional vitiligo skin and we show that this process is part of a broader metabolic program and may act as a compensatory/protective response towards the intrinsic metabolic vulnerability.

## Results

### Non-lesional vitiligo melanocytes show increased expression of autophagic markers

**Fig. 1:**
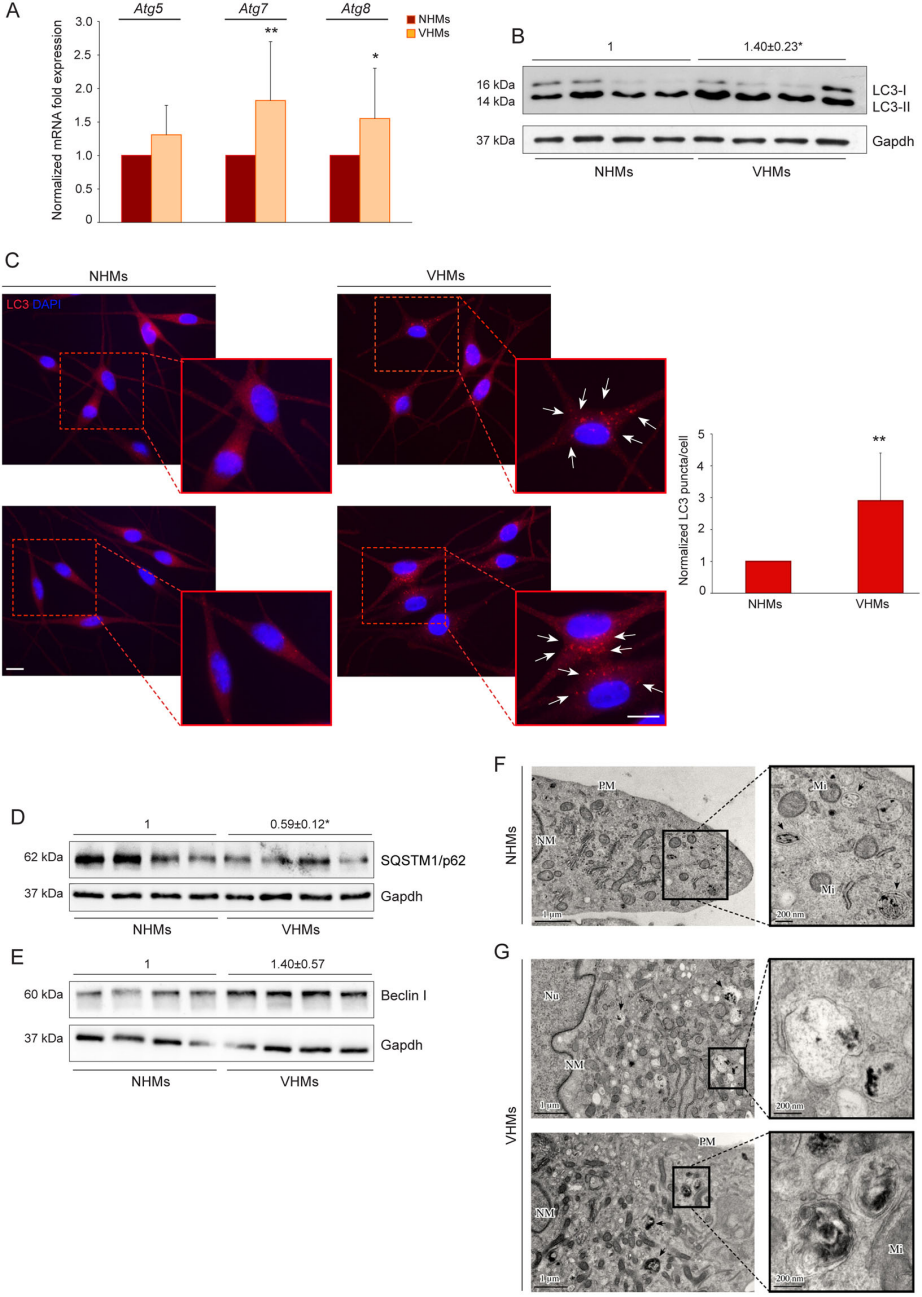
Autophagy process is induced in non-lesional vitiligo melanocytes. **A** mRNA transcripts of autophagy genes *Atg5, Atg7, Atg8* (NHMs *n* = 7 and VHMs *n* = 7); **B** Western blot with average value of densitometric analysis of LC3-II/I expression on vitiligo lysates normalized on control ones. A representative blot is shown (NHMs *n* = 4 and VHMs *n* = 4); **C** Immunofluorescence analysis of LC3 (red) and corresponding quantitative analysis of the number of LC3 positive puncta per cell. Nuclei were counterstained with DAPI. Red boxes show the enlarged view of the selected dashed areas. The arrows point at LC3 positive dots. Quantification of positive puncta per cell is expressed as fold change relative to NHMs value, which was set as 1 by definition (*n*= at least 150 cells per group). Scale bars 10 μm; **D**, **E** Western blot of SQSTM1/p62 and Beclin I expression and corresponding densitometric analyses on vitiligo lysates normalized on control ones (NHM *n* = 4; VHM *n* = 4); **F** TEM representative images of normal human melanocytes (NHMs) and **G** non-lesional vitiligo melanocytes (VHMs). Black arrows in **F** point at different stages of melanosomes (see magnification). Black arrows in **G** indicate degradative autophagic vacuoles containing melanin or melanosomes (see magnification) (NHM *n* = 3; VHM *n* = 3); TEM micrographs, uranyl acetate/lead citrate; Nu, Nucleus; NM, nuclear membrane; PM, plasma membrane; Mi, Mitochondrion. *Atg5*, autophagy related 5; *Atg7*, autophagy related 7; *Atg8*, autophagy related 8; LC3, (microtubule-associated protein 1) light chain 3; NHMs, normal human melanocytes; SQSTM1/p62, sequestosome-1; VHMs, vitiligo human melanocytes.

To investigate the possible involvement of autophagy in vitiligo, we initially determined the relative mRNA abundance of some *Atg* transcripts (*Atg5, Atg7, Atg8*) by quantitative reverse-transcriptase PCR (Fig. 1A). The expression levels of *Atg7* and *Atg8* were significantly higher in non-lesional vitiligo melanocytes (VHMs) than in control cells (NHMs), while the level of *Atg5* showed a tendency to increase, although this was not statistically significant. Elevated levels of autophagy were also demonstrated using the well-established marker for phagophores and autophagosomes LC3^17,18^ whose LC3-II form was significantly enhanced in vitiligo melanocytes as determined by western blot analysis (Fig. 1B). Immunofluorescence staining accompanied by the quantification of LC3 positive puncta per cell confirmed the higher expression in vitiligo (Fig. 1C). Furthermore, a statistically significant decrease in the autophagy adaptor protein SQSTM1/p62 levels was observed (Fig. 1D). We also found that Beclin I, a protein involved in both the signaling pathway activating autophagy and the initial step of autophagosome formation, showed a tendency to be upregulated in vitiligo melanocytes, although this increase was not significant (Fig. 1E). The presence of autophagy was also confirmed by transmission electron microscopy (TEM). The ultrastructural analysis revealed the presence of melanosomes at different stages in the cytoplasm of NHMs (Fig. 1F, see magnification). VHMs showed numerous degradative autophagic vacuoles (black arrows) containing melanin or melanosomes (Fig. 1G, see magnification).

### AMPK-mTORC1 signaling is responsible for autophagy activation in vitiligo melanocytes

**Fig. 2:**
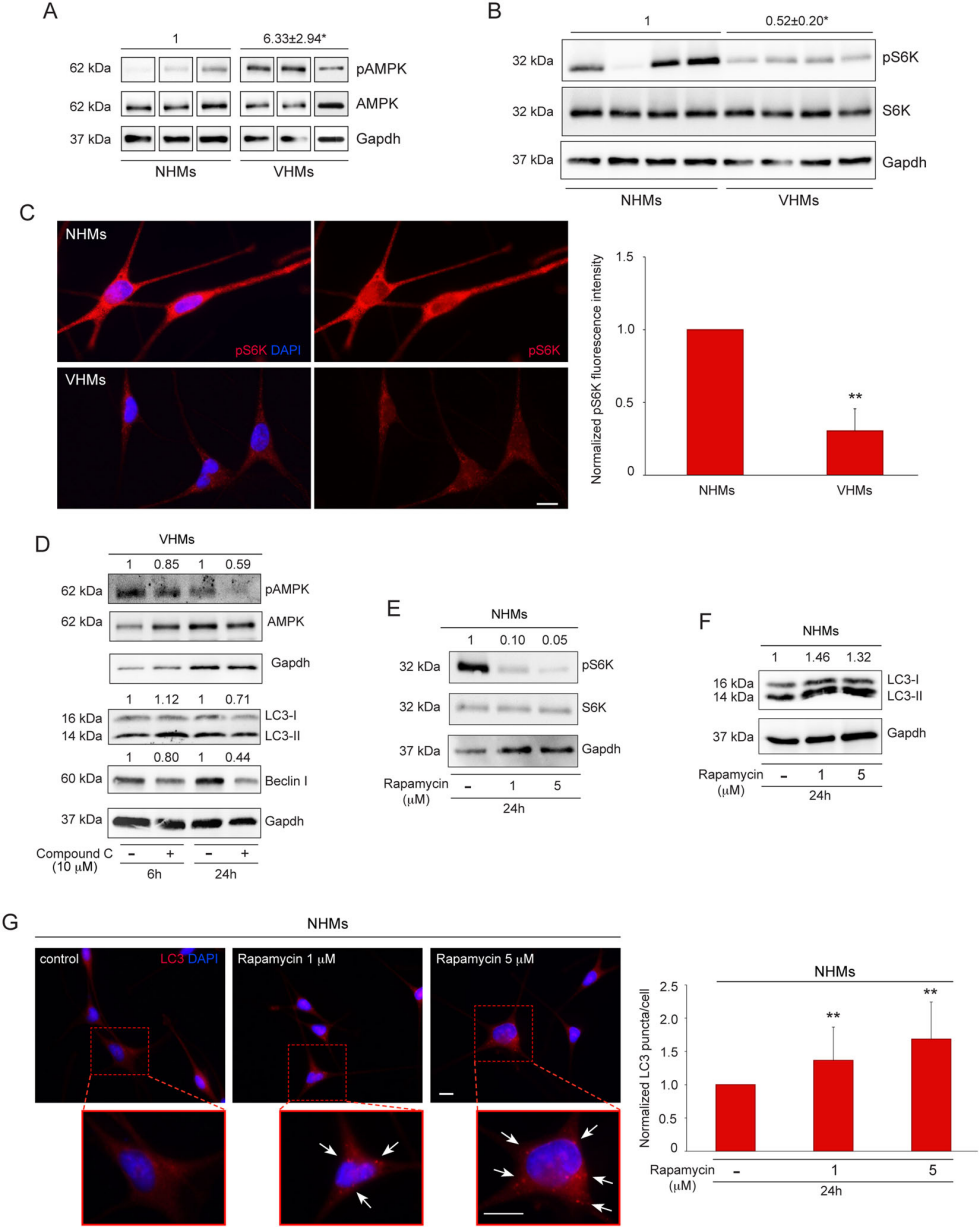
Metabolic alterations trigger autophagy process through AMPK-mTORC1 signaling. **A** Western blot and normalized densitometric analysis of pAMPK expression in normal and non-lesional vitiligo melanocytes (NHMs *n* = 3; VHMs *n* = 3); **B** Western blot of pS6K expression with average value of normalized densitometric analyses on cell lysates (NHMs *n* = 4; VHMs *n* = 4); **C** Immunofluorescence staining and corresponding normalized fluorescence intensity for pS6K (red) in normal and vitiligo melanocytes. Nuclei were counterstained with DAPI. A representative normal melanocyte culture paired with a vitiligo melanocyte one is shown. Scale bar: 10 μm; **D** Western blot of pAMPK, AMPK, LC3-II/I, and Beclin I with normalized densitometric analysis on vitiligo melanocytes treated or not with Compound C (10 μM) for 6 and 24 h. Representative blots of three independent experiments are shown; **E**, **F** Western blot of pS6K and LC3-II/I expression and corresponding values of normalized densitometric analysis on cell lysates of normal melanocytes after treatment with the mTORC1 inhibitor rapamycin (1 and 5 μM) for 24 h. A representative blot of three independent experiments is shown; **G** Immunofluorescence staining for LC3 and corresponding quantitative analysis of the number of LC3 positive puncta per cell (*n* = at least 150 cells per group) in untreated and rapamycin-treated normal melanocytes for 24 h. Nuclei were counterstained with DAPI. Red boxes show the enlarged view of the selected dashed areas. The arrows point at LC3 positive dots. Scale bars: 10 μm. A representative normal melanocyte culture is shown. pAMPK, phospho-adenosine monophosphate (AMP)-activated protein kinase; LC3, (microtubule-associated protein 1) light chain 3; NHMs, normal human melanocytes; pS6K, Phospho S6 kinase; VHMs, vitiligo human melanocytes.

We recently demonstrated that melanocytes obtained from normally pigmented vitiligo skin exhibit defects in mitochondrial metabolism^2^. Since alterations in cellular metabolic status can induce autophagy through activation of the energy sensor AMPK and inhibition of mTORC1 pathway^11,12^, we investigated the activity of both in non-lesional vitiligo melanocytes. The results showed that the expression of AMPK phosphorylated on Thr172 (pAMPK), a marker of its activation, was upregulated (Fig. 2A), whereas the phosphorylation level of the downstream target of mTORC1, S6 kinase (pS6K), was reduced compared to control melanocytes, as revealed by western blotting (Fig. 2B) and immunofluorescence accompanied by staining intensity image analysis (Fig. 2C). The above results suggest that in vitiligo, autophagy is regulated by AMPK activation and subsequent mTORC1 pathway inhibition. To deeper substantiate the involvement of AMPK-mTORC1 signaling into the catabolic process, we determined the levels of LC3-II form and Beclin I in vitiligo melanocytes after inhibiting AMPK activation by Compound C. We observed a time-dependent decrease of phosphorylated AMPK, which was associated with reduced levels of both proteins after 24 h (Fig. 2D). In parallel, treatment of normal melanocytes with the mTORC1 inhibitor rapamycin down-modulated pS6, indicating the efficacy of its action (Fig. 2E) and it increased LC3 expression, as assessed by western blot (Fig. 2F) and immunofluorescence (Fig. 2G) analyses, proving that autophagy goes through mTORC1 pathway inhibition in vitiligo.

### Autophagy induction may be ascribed to impaired energy metabolism in non-lesional vitiligo melanocytes

**Fig. 3:**
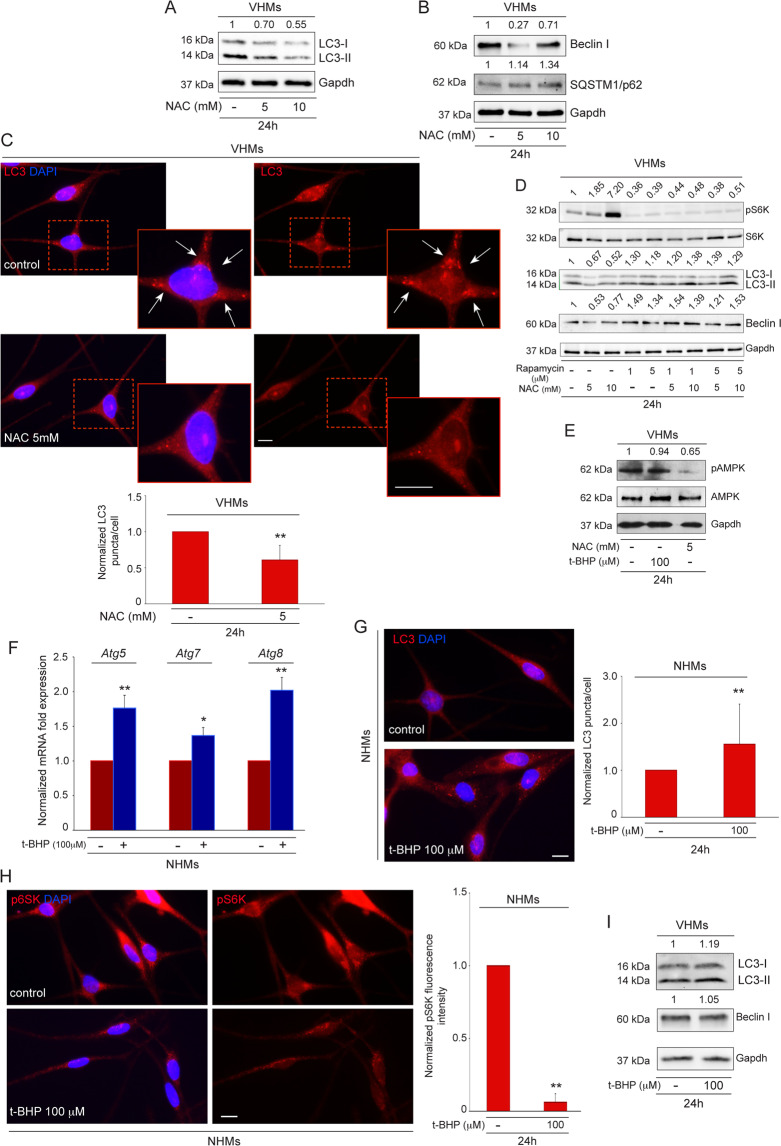
Autophagy can be ascribed to impaired energy metabolism in Vitiligo. **A** Western blot of LC3-II/I with corresponding normalized densitometric values on lysates of vitiligo melanocytes after treatment with NAC (5 and 10 mM) for 24 h. A representative blot of three independent experiments is shown; **B** Western blot of Beclin I and SQSTM1/p62 with normalized densitometric analysis on vitiligo melanocytes treated with NAC (5 and 10 mM) for 24 h. Representative blots of three independent experiments are shown; **C** Immunofluorescence of LC3 expression (red) and corresponding quantitative analysis of the number of LC3 positive puncta per cell (n = at least 150 cells per group) in vitiligo melanocytes treated or not with NAC (5 mM) for 24 h. Nuclei were counterstained with DAPI. Boxes show the enlarged view of the selected cells. The arrows point at LC3 positive dots. Scale bars: 10 μm. A representative vitiligo melanocyte culture is shown; **D** Western blot of pS6K, LC3-II/I and Beclin I with corresponding normalized densitometric values on lysates of vitiligo melanocytes pretreated with Rapamycin (1 and 5 uM) for two hours followed by NAC-treatment (5 and 10 mM) for 24 h. A representative blot of three independent experiments is shown; **E** Western blot of pAMPK with corresponding values of normalized densitometric analysis on cell lysates of vitiligo melanocytes after treatment with NAC (5 mM) and t-BHP (100uM) for 24 h. A representative blot of three independent experiments is shown; **F** Normalized mRNA transcripts of autophagy genes *Atg5, Atg7, Atg8* following t-BHP treatment (100 uM) for 24 h in normal melanocytes (NHM *n* = 3); **G** Immunofluorescence analysis of LC3 (red) and corresponding quantitative analysis of the number of LC3 positive puncta per cell (*n* = at least 150 cells per group) in t-BHP-treated (100 uM for 24 h) normal melanocytes. Nuclei were counterstained with DAPI. Scale bar: 10 μm. A representative normal melanocyte culture treated with t-BHP is shown; **H** Immunofluorescence and corresponding normalized fluorescence intensity of pS6K (red) in t-BHP-treated (100 uM for 24 h) normal melanocytes. Nuclei were counterstained with DAPI. A representative normal melanocyte culture treated with t-BHP is shown. Scale bar: 10 μm; **I** Western blot of LC3-II/I and Beclin I with normalized densitometric analysis on vitiligo melanocytes treated with t-BHP (100 uM) for 24 h. Representative blots of three independent experiments are shown. pAMPK, phospho-adenosine monophosphate (AMP)-activated protein kinase; *Atg5*, autophagy related 5; *Atg7*, autophagy related 7; *Atg8*, autophagy related 8; LC3-I/II, (microtubule-associated protein 1) light chain 3; NAC, N-acetyl-L-cystein; NHMs, normal human melanocytes; SQSTM1/p62, sequestosome-1; t-BHP, tert-butyl hydroperoxide; pS6K, Phospho S6 kinase; VHMs, vitiligo human melanocytes.

Autophagy is strictly connected to metabolic homeostasis. Consequently, it is plausible that, in our system, autophagy induction could be related to redox unbalance caused by defects in cell metabolism. Therefore, the expression of autophagic markers was analyzed in vitiligo melanocytes treated with N-acetyl-L-cysteine (NAC), which increases the concentration of the antioxidant glutathione and enhances energy metabolism by preventing mitochondrial potential loss, regulating respiratory chain enzymes, and decreasing the ADP/ATP ratio^19,20^. *Atg5* and *Atg7* mRNAs decreased after NAC treatment with a reduction of protein expression mainly evident for Atg7 (Fig S1A, B). Western blot analyses also demonstrated that LC3-II/I and Beclin I protein levels were significantly lower in the presence of NAC (Fig. 3A, B), concurrently with the upregulation of SQSTM1/p62 protein expression (Fig. 3B). In line with these data, parallel immunofluorescence analysis revealed the ability of NAC to decrease LC3 staining intensity in treated cells in comparison to control (Fig. 3C). The link between the reduction of autophagy and mTORC1 pathway activation was demonstrated by the increase of S6K phosphorylation in vitiligo melanocytes after treatment with NAC, as revealed through immunofluorescence analysis and quantitative measurement of the staining intensity (Fig. S1C). Moreover, we demonstrated that NAC treatment was unable to increase pS6K expression in vitiligo cells pretreated with Rapamycin inhibitor (Fig. 3D). Accordingly, the co-treatment did not cause any down-modulation of LC3-II/I and Beclin I expression (Fig. 3D) further confirming the important role of mTORC1 pathway in decreasing NAC-induced autophagy.

In line with these results, also the expression level of phosphorylated AMPK decreased in vitiligo melanocytes treated with NAC (Fig. 3 E).

Interestingly, NAC supplementation resulted in significant increase of ATP production (Fig. S1D) highlighting that autophagy reduction by NAC in vitiligo melanocytes may reflect changes in cell energy metabolism.

To corroborate the biological significance of our results, we asked if the activation of autophagy could be recapitulated in normal human melanocytes treated with tert-butyl hydroperoxide (t-BHP) to induce mitochondrial dysfunction^21^. Treatment with t-BHP was able to induce an overexpression of both *Atg* mRNAs (Fig. 3F) and LC3 protein in normal melanocytes (Fig. 3G). Moreover, the level of phosphorylated S6K was reduced in cells treated with t-BHP, confirming that stress-induced autophagy goes through mTORC1 pathway inhibition in vitiligo melanocytes (Fig. 3H). Accordingly, ATP production decreased in t-BHP-treated normal melanocytes (Fig. S1E). Interestingly, tert-butyl hydroperoxide did not further increase autophagy process in vitiligo melanocytes, as showed by LC3-II/I and Beclin I protein expression (Fig. 3I). In line with these results, pAMPK level did not increase after t-BHP treatment either (Fig. 3E), suggesting that in vitiligo, the autophagic process is activated at such a level that it does not respond to additional stimuli.

### Autophagy in non-lesional vitiligo fibroblasts

**Fig. 4:**
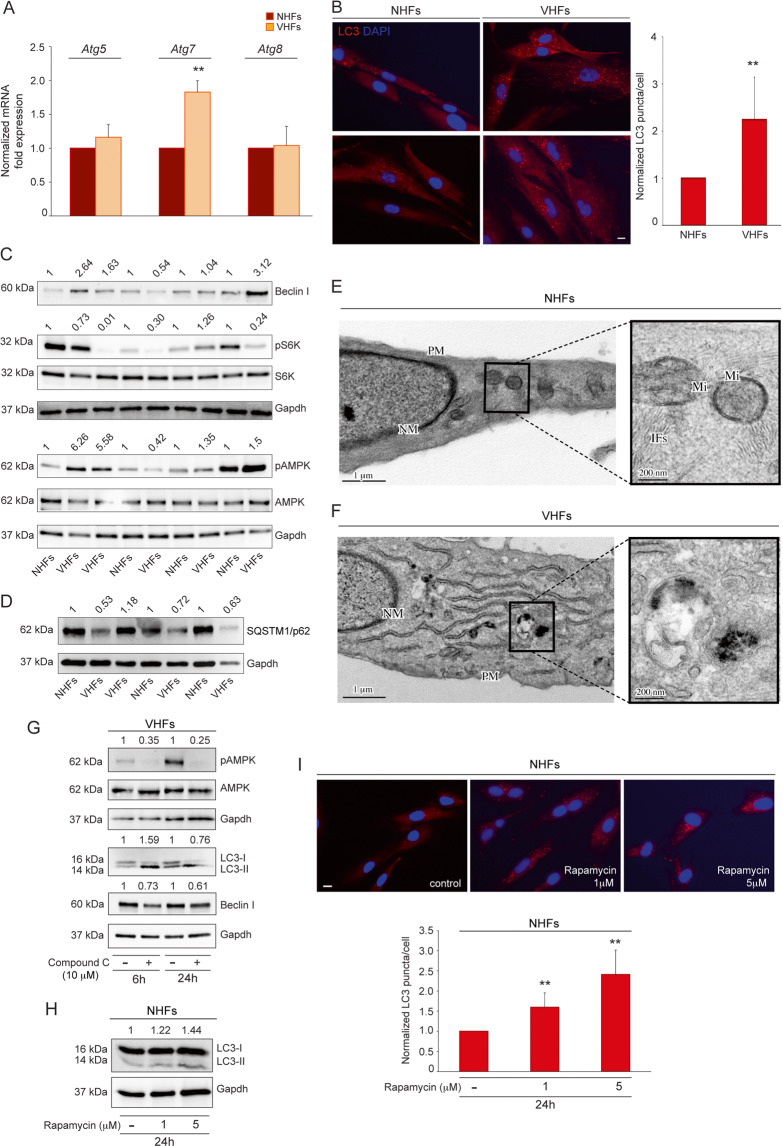
Non-lesional vitiligo fibroblasts display a tendency to incur in the autophagic process. **A** *Atg5, Atg7, Atg8* mRNA level evaluated by qRT-PCR in normal and non-lesional vitiligo fibroblasts (NHFs *n* = 5 and VHFs *n* = 5); **B** Immunofluorescence analysis of LC3 (red) and corresponding quantitative analysis of the number of LC3 positive puncta per cell. Nuclei were counterstained with DAPI. Quantification of positive puncta per cell is expressed as fold change relative to NHFs value, which was set as 1 by definition (*n* = at least 150 cells per group). Scale bar: 10 μm; **C** Western blot of Beclin I, pS6 and pAMPK with corresponding densitometric analyses on lysates of vitiligo fibroblasts normalized on control ones (NHFs *n* = 4 and VHFs *n* = 5); **D** SQSTM1/p62 with corresponding densitometric analyses on lysates of vitiligo fibroblasts normalized on control ones (NHFs *n* = 3 and VHFs *n* = 4); **E** Ultrastructural features of normal human fibroblasts with typical spindle-like shape and cytoplasm with a few rough endoplasmic reticulum cisternae and intracellular filaments (see magnification) (NHF *n* = 3); **F** TEM representative image of non-lesional vitiligo fibroblasts characterized by higher cytoplasmatic complexity with a prominent Golgi apparatus, many cytoplasmic vesicles, well-developed rough endoplasmic reticulum and intracellular filament bundles localized near the plasma membrane. Into the cytoplasm autophagic vacuoles are also identifiable (see magnification) (VHF *n* = 3); TEM micrographs, uranyl acetate/lead citrate; NM, nuclear membrane, PM, plasma membrane; Mi, Mitochondrion; IFs: Intermediate filaments. **G** Western blot of pAMPK, LC3II/I, and Beclin I with normalized densitometric analysis on vitiligo fibroblasts treated or not with Compound C (10 uM) for 6 and 24 h. Representative blots of three independent experiments are shown; **H** Western blot of LC3-II/I expression and corresponding values of normalized densitometric analysis on cell lysates of normal fibroblasts after treatment with the mTORC1 inhibitor rapamycin (1 and 5 μM) for 24 h. A representative blot of three independent experiments is shown; **I** Immunofluorescence staining and corresponding quantitative analysis of the positive number of LC3 puncta in untreated and rapamycin-treated normal fibroblasts for 24 h (*n* = at least 150 cells per group). Nuclei were counterstained with DAPI. Scale bar: 10 μm. A representative normal fibroblast culture treated with rapamycin is shown. *Atg7*, autophagy related 7; *Atg8*, autophagy related 8; *Atg5*, autophagy related 5; p-AMPK, phospho-adenosine monophosphate (AMP)-activated protein kinase; LC3-I/II, (microtubule-associated protein 1) light chain 3; NHFs, normal human fibroblasts; SQSTM1/p62, sequestosome-1; qRT-PCR, quantitative real time reverse transcriptase-PCR; VHFs, vitiligo human fibroblasts.

We recently demonstrated that also fibroblasts obtained from non-lesional skin of vitiligo patients show features resembling a senescent phenotype^22^. Following the concept that vitiligo should be considered a disease of the entire skin rather than being melanocyte-specific, the analysis of autophagy was extended to non-lesional vitiligo fibroblasts. We demonstrated that, although *Atg5* and *Atg8* mRNAs were not upmodulated (Fig. 4A), vitiligo fibroblasts showed a tendency to exhibit elevated autophagy, as assessed by increases of *Atg7* gene expression (Fig. 4A), LC3 (Fig. S2A, Fig. 4B), and Beclin I production (Fig. 4C) concurrently with the down-modulation of SQSTM1/p62 (Fig. 4D). Moreover, similar to vitiligo melanocytes, autophagy induction was reflected in the inhibition of mTORC1, as shown by down-modulation of pS6K (Fig. 4C, Fig. S2B) and by increase of phosphorylated AMPK (Fig. 4C).

Parallel ultrastructural analysis of normal human fibroblasts showed a typical spindle-like shape and a cytoplasm with a few rough endoplasmic reticulum cisternae and intracellular filaments (Fig. 4E, see magnification). Non-lesional vitiligo fibroblasts appeared characterized by higher cytoplasmatic complexity and autophagic vacuoles were also identifiable into the cytoplasm (Fig. 4F, see magnification).

Furthermore, as for melanocytes, we treated the cells with Compound C and the results proved that AMPK inactivation negatively influences autophagy process also in vitiligo fibroblasts, at 24 h of treatment (Fig. 4G). The induction of LC3II/I observed at 6 h may be ascribed to an AMPK inhibition-independent downregulation of Akt/mTOR pathway, as previously reported. Moreover, the effect of Compound C has been demonstrated to be dose-, cell type- and/or context dependent^23^. In parallel, the mTORC1 inhibitor rapamycin increased LC3 expression in normal fibroblasts, as assessed by western blotting and immunofluorescence analyses (Fig. 4H, I).

### Autophagy inhibition exacerbates adverse metabolic effects in non-lesional vitiligo cells

**Fig. 5:**
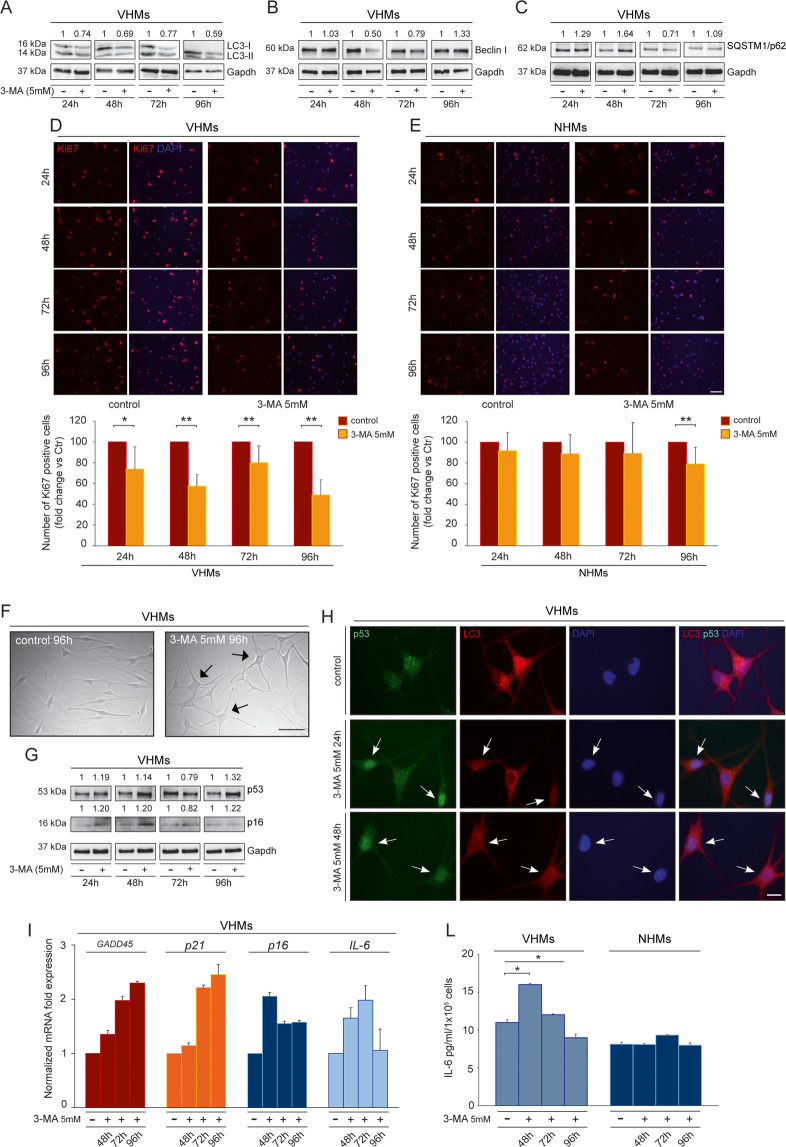
Inhibition of autophagy increases adverse metabolic effects in non-lesional vitiligo cells. **A**, **B**, **C** Western blot of LC3-II/I, Beclin I and SQSTM1/p62 with corresponding normalized densitometric analysis on lysates of vitiligo melanocytes untreated or treated with 3MA (5 mM) for 24–48–72–96 h. Representative blots of three independent experiments are shown; **D** Immunofluorescence staining for Ki67 (red) and the corresponding percentage of Ki67 positive cells in vitiligo melanocytes before and after treatment with 3-MA (5 mM) for 24–48–72–96 h (*n* = at least 700 cells per group). Nuclei were counterstained with DAPI. Scale bar: 50 μm; **E** Immunofluorescence analysis and percentage of positive cells for Ki67 (red) in normal melanocytes before and after treatment with 3-MA (5 mM) for 24-48-72-96 h (*n* = at least 700 cells per group). Nuclei were counterstained with DAPI. Scale bar: 50 μm; **F** Morphological analysis of vitiligo melanocytes untreated and treated with 3-MA (5 mM) for 96 h. Arrows point at cells showing an enlarged morphology. Scale bar: 50 μm; A representative vitiligo melanocyte culture treated with 3-MA is shown. **G** Western blot of p53 and p16 with corresponding normalized densitometric analysis on lysates of vitiligo melanocytes untreated or treated with 3MA (5 mM) for 24–48–72–96 h. Representative blots of three independent experiments are shown; **H** Double immunofluorescence analysis of p53 (green) and LC3 (red) expression in vitiligo melanocytes untreated and treated with 3MA (5 mM) for 24 and 48 h. Nuclei were counterstained with DAPI. The arrows point at cells showing p53 nuclear translocation. Scale bar: 10 μm; A representative vitiligo melanocyte culture treated with 3-MA is shown. **I** mRNA transcripts of *Gadd45*, *p21*, *p16* and *IL-6* evaluated by qRT-PCR in vitiligo melanocytes untreated and treated with 3MA (5 mM) for 48–72–96 h (VHMs *n* = 3); **L** IL-6 quantification by ELISA in vitiligo and normal melanocytes untreated and treated with 3-MA (5 mM) for 48-72-96 h (VHMs *n* = 3 and NHMs *n* = 3). *Gadd45*, growth arrest and DNA damage-inducible 45; IL-6, interleukin 6; LC3-I/II, (microtubule-associated protein 1) light chain 3; NHMs, normal human melanocytes; 3-MA, 3-methyl adenine; VHMs, vitiligo human melanocytes.

Autophagy is an important cellular program with a potential “double faced” role that can promote either cell survival or cell death. Hence, we explored if autophagy in vitiligo may be part of a response to a broader metabolic shift, and whether it tends to maintain cell survival or whether it represents a degenerative mechanism through autophagy-mediated programmed cell death. We, therefore, inhibited autophagy in both vitiligo melanocytes and fibroblasts using 3-methyl adenine (3-MA) that blocks the activity of hVps34, which is essential for phosphatidylinositol 3-phosphate production (PI3P) during the early stages of autophagosome assembly^24^. Our results revealed that autophagy genes, LC3-II/I and Beclin I expression decreased after treatment with 3-MA (Fig. S3A, Fig. 5A, B) concurrently with the up-modulation of SQSTM1/p62 (Fig. 5C). 3-MA modulating autophagy may depend on different conditions^25^, which can account for the variable protein expression observed at the time points tested.

Autophagy reduction was associated with decreased proliferation in vitiligo melanocytes, as determined by immunofluorescence analysis using an anti-Ki67 antibody to identify cycling cells (Fig. 5D). Quantitative analysis showed a significant decrease in the number of proliferating cells after 5 mM 3MA treatment at all time points evaluated (Fig. 5D). Reduction in the rate of cell growth was also confirmed by MTT assay (Fig S3B). By contrast, the proliferation of normal melanocytes was reduced in response to autophagy inhibition only after 96 h of treatment. Such decrease appeared also less severe in comparison to that observed for vitiligo melanocytes (Fig. 5E). In addition, vitiligo melanocytes underwent changes in cell morphology characterized by an enlarged cytoplasm (Fig. 5F). Interestingly, such cellular changes were accompanied by an increased p53 expression (Fig. 5G) and by its nuclear translocation in treated cells (Fig. 5H) in comparison to control, where the signal appeared mainly distributed throughout the cytoplasm, as previously reported^26^. Moreover, double immunofluorescence analysis with anti-p53 and anti-LC3 antibodies showed a reduction of LC3-positive dots in cells characterized by nuclear translocation of p53 (Fig. 5H, arrows), suggesting its activation when autophagy was effectively inhibited. As further proof of p53 activation, a higher expression of its target gene GADD45, as well as of p21, p16, and IL-6 was detected in 3MA-treated vitiligo melanocytes (Fig. 5I). Moreover, p16 was also up-modulated as protein (Fig. 5G) and a transient enhanced release of IL-6 was even observed at the protein level by ELISA assay on culture supernatants of vitiligo melanocytes, particularly after 48 h of treatment with 3-MA (Fig. 5L). By contrast, normal melanocytes did not exhibit a significant production of IL-6 (Fig. 5L).

**Fig. 6:**
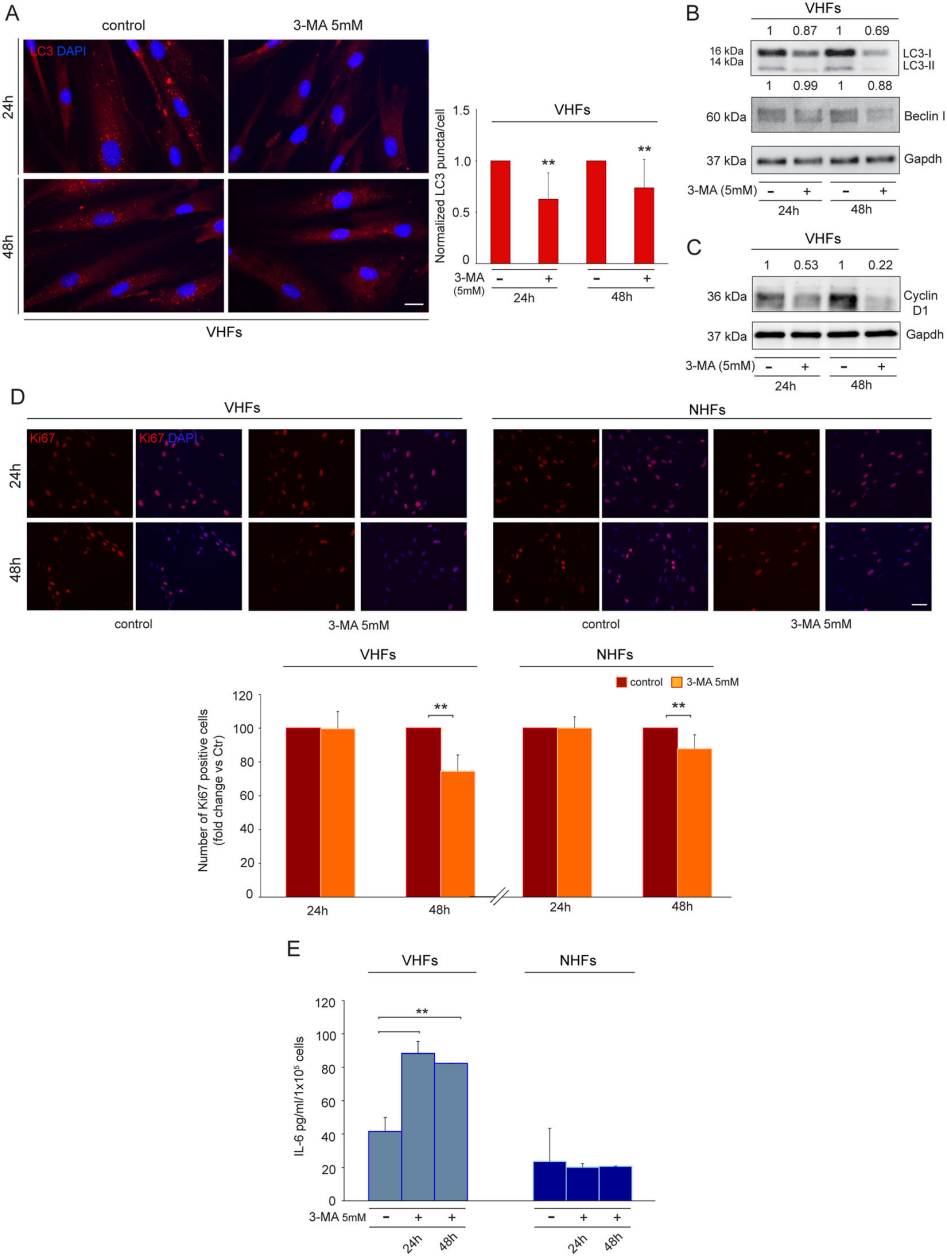
Suppression of autophagy further impairs Vitiligo fibroblasts. **A** Immunofluorescence analysis and corresponding quantitative analysis of the number of positive LC3 puncta on vitiligo fibroblasts untreated or treated with 3MA (5 mM) for 24 and 48 h (*n* = at least 150 cells per group). Nuclei were counterstained with DAPI. Scale bar: 20 μm. A representative vitiligo fibroblast culture treated with 3-MA is shown; **B**, **C** Western blot of LC3-II/I, Beclin I, and Cyclin D1 and corresponding densitometric analysis on vitiligo lysates normalized on control ones after treatment with 3-MA (5 mM) for 24 and 48 h. Representative blots of three independent experiments are shown. **D** Immunofluorescence staining of Ki67 (red) and corresponding percentage of Ki67 positive vitiligo and normal fibroblasts treated or not with 3-MA (5 mM) for 24 and 48 h (*n* *=* at least 700 cells per group). Nuclei were counterstained with DAPI. Scale bar: 50 μm; **E** IL-6 quantification by ELISA in vitiligo and normal fibroblasts untreated and treated with 3-MA (5 mM) for 24 and 48 h (VHFs *n* = 3 and NHFs *n* = 3). *IL-6*, interleukin 6; LC3-I/II, (microtubule-associated protein 1) light chain 3; 3-MA, 3-methyl adenine; qRT-PCR, quantitative real-time reverse transcriptase-PCR; VHFs, vitiligo human fibroblasts.

Similar to melanocytes, also vitiligo fibroblasts exhibited a modulation of cell-cycle regulators by 3-MA treatment. The inhibition of autophagy, which is demonstrated by diminished LC3 signal intensity (Fig. 6A), LC3-II/I and Beclin I expression (Fig. 6B), caused a decrease of Cyclin D1 (Fig. 6C), indicating a functional cell cycle arrest. To further analyze the effect of 3-MA on vitiligo fibroblast growth, parallel immunofluorescence analysis employing the proliferation marker Ki67 demonstrated a decrease in percentage of cycling fibroblasts after 3-MA, which appeared significant at 48 h of treatment. Similar to melanocytes, the decrease in cell proliferation resulted less prominent in normal fibroblasts (Fig. 6D).

Furthermore, 3-MA treated fibroblasts expressed higher levels of secreted IL-6 protein in comparison to control cells (Fig. 6E).

Based on these results, the cell-cycle and the morphological and functional characteristics resemble a senescent associated secretory phenotype and suggest that the inhibition of autophagy exacerbates deleterious metabolic effects on vitiligo melanocytes and fibroblasts.

## Discussion

The key role of autoimmunity, through the involvement of both humoral and cellular immunity, in the pathogenesis of vitiligo has been clearly accepted. Nevertheless, immune responses are just the “tip of the iceberg”. Recently, mitochondrial dysfunction, which results in altered electron transport chain as well as in more ROS generation and less ATP production, has emerged as a further mechanism contributing to the disease. However, how vitiligo melanocytes coordinate the response to the impairment of energetic metabolism and how these metabolic alterations trigger intracellular signaling that eventually may lead to an immune response are key issues.

Autophagy is a lysosomal degradation process that is required for the survival of cells under metabolic stress. In vitiligo context, it may be part of a broader metabolic program that promotes maintenance over growth by mediating an adaptive response directed towards rebalancing metabolic supply. To date, all the experiments carried out on the role of autophagy in vitiligo were focused on cutaneous lesional areas of patients. In this study, we went further in providing the evidence that autophagy occurs in melanocytes and fibroblasts from non-lesional skin, as a result of metabolic surveillance response. More precisely, we revealed that induction of autophagy exerts a protective role against the intrinsic metabolic stress occurring in vitiligo. Consistent with this, we showed that the N-acetyl cysteine, by enhancing energy metabolism, down modulates the expression level of autophagic markers in vitiligo melanocytes. Vice versa, treatment with the pro-oxidant tert-butyl hydroperoxide is able to induce an overexpression of both *Atg* mRNAs and LC3 protein in normal melanocytes. Consequently, we supposed that induction of autophagy is linked with metabolic injury in vitiligo.

Among the macromolecular signaling complexes controlling autophagy AMPK, that is activated by an increase in AMP/ATP ratio, works as a fuel-sensing enzyme and may induce autophagy. In contrast, mTORC1 is a stress-sensitive kinase complex inhibiting the process^27^. Impaired energy levels lead to dephosphorylation of mTORC1 sites with the consequent induction of autophagy cascade^27^. Furthermore, mTORC1 is also a transcriptional regulator of autophagy since it inactivates some transcription factors that are involved in lysosomal biogenesis and function and that sustain autophagy activity^28^. Interestingly, under stress conditions, the inhibition of the mTORC1 pathway stimulates nuclear translocation of the microphthalmia-associated transcription factor (Mitf), which is essential at multiple stages of the melanocyte life cycle. Within the nucleus, Mitf is implicated in the transcription of autophagy genes^29^.

We previously demonstrated that melanocytes and fibroblasts from vitiligo patients displayed intrinsic alterations related to metabolic impairment that, when persistent, may lead to a senescent phenotype^1,22^. Autophagy is activated at senescence, as shown for normal senescent epidermal keratinocytes^30^, normal senescent fibroblasts^31^, long-term cultured T lymphocytes^32^ and some tumor cell lines re-induced into senescence after drug treatments^33,34^.

Here we show that the level of autophagic activity correlates with defects in mitochondrial metabolism and senescent phenotype of vitiligo melanocytes and fibroblasts. This opened the question of what is the role of autophagy in vitiligo. Remarkably, this process may contribute to the preservation of cellular energy balance by recycling of glucose, amino acids and lipids in cells^35–37^, and recycled amino acids can enter into Krebs cycle to produce ATP^38,39^.

We demonstrated that inhibition of autophagy worsened the senescence phenotype of vitiligo melanocytes, as revealed by an increased expression and nuclear translocation of p53, a higher expression of its target gene GADD45 and by elevated levels of p21 and p16. In parallel, 3MA-treated vitiligo fibroblasts showed a decreased Cyclin D1 expression, thus suggesting an arrest in cell cycle progression. Moreover, the inhibition of autophagy can exacerbate the progress of some inflammatory and autoimmune diseases through the production of inflammatory cytokines^40^. Accordingly, we also detected a significant induction of IL-6 expression in both vitiligo cell populations after being treated with 3-MA.

As a consequence of these observations, our results suggest that autophagy may be an adaptive response to continuous metabolic alterations and that its induction may result in a protective attempt to antagonize degenerative processes and to enhance survival in normal appearing vitiligo skin, where melanocytes and fibroblasts are already prone to premature senescence.

## Materials and methods

### Skin biopsies and cell cultures

Specimens were collected from non-lesional gluteal skin areas of vitiligo subjects with non-segmental disease observed in the San Gallicano Dermatological Institute. At the time of patient enrollment, none of the subjects had received either local or systemic therapy for at least 5 months and only those with stable vitiligo and with body surface area involvement less than 10% were included. Normal human skin samples were obtained from healthy volunteers subjected to plastic surgery. The study was approved by the Medical Ethical Committee of the San Gallicano Dermatological Institute and it was conducted according to the Declaration of Helsinki principles. Participants gave their written informed consent.

After mechanical dissection of skin biopsies, the epidermis was separated from the dermis by digestion with dispase 0·1 mg mL^−1^ (Gibco; Invitrogen). Epidermal sheets were incubated in a solution of trypsin 0.05% and ethylenediamine tetraacetic acid 0.02% in PBS (Euroclone Ltd, Wetherby, UK), under continuous and gentle shaking in order to separate cellular elements. Primary cultures of melanocytes were selectively grown in M254 medium with Human Melanocytes Growth Supplement (HMGS; Life Technologies Italia, Monza, Italy) and antibiotics. Seven vitiligo and control melanocyte cultures were evaluated and experiments carried out from II to VI passages. Primary dermal fibroblasts were collected from the dermis that was incubated with collagenase 0·35% (Gibco; Invitrogen) and they were cultured in Dulbecco’s modified medium (EuroClone S.p.A., Milan Italy) with 10% fetal bovine serum (Hyclone Laboratories, South Logan, UT, USA) and antibiotics. The experiments were performed using five vitiligo and control fibroblasts from short-term cultures (II–X cell culture passages). All cells were screened for mycoplasma.

### Cell cultures treatments

Vitiligo melanocytes were treated with the AMPK inhibitor (10uM) Compound C (Sigma Aldrich, Milan, Italy) for 6 and 24 h. Normal human melanocytes were incubated with the mTORC1 inhibitor rapamycin (1 and 5 μM) (Sigma Aldrich, Milan, Italy) for 24 h. To check the role of intracellular oxidative stress in inducing autophagy process, vitiligo melanocytes were cultured in M254 containing HMGS and treated with N-acetyl-L-cystein (5 and 10 mM) (Sigma Aldrich, Milan, Italy) for 24 h. In parallel, normal human melanocytes were grown in M254 containing HMGS and exposed to 100μM of tert-butyl hydroperoxide (Sigma Aldrich) for 24 h. To inhibit autophagy, vitiligo melanocytes were grown in M254 containing HMGS and treated with the autophagy inhibitor 3-methyl adenine (5 mM) (Sigma Aldrich) for 24-48-72-96 h whereas vitiligo fibroblasts were cultured in DMEM with 10% FBS and incubated with the inhibitor 3-methyl adenine (5 mM) (Sigma Aldrich) for 24 and 48 h.

### ATP determination

The intracellular level of ATP was measured using a commercial ATP Colorimetric/Fluorometric Assay Kit (BioVision) according to the manufacturer’s instructions. The results were reported as fold change relative to untreated cells value, which was set as 1 by definition.

### MTT Assay

Normal and vitiligo melanocytes, treated with the autophagy inhibitor 3-methyl adenine as indicated, were then incubated with 3-(4,5-dimethyl-2-thiazolyl)-2,5-diphenyl-2H-tetrazolium bromide (MTT) (1 mg/mL) for 2 h at 37 °C, and lysed in dimethyl sulfoxide (DMSO). The absorbance at 570 nm was measured by a spectrophotometer DTX880 Multimode Detector (Beckman Coulter srl., Milano, Italy). The results are expressed as fold change relative to untreated cells value (%).

### RNA extraction and Quantitative real-time PCR

Total RNA was isolated by using the Aurum^TM^ Total RNA Mini kit (Bio-Rad Laboratories Srl, Milan, Italy). The RNA yields, purity, and quality in terms of lack of degradation were determined by OD 260/280 absorbance measurements. cDNA was synthesized from 1 μg of total RNA using the RevertAid^TM^ First Strand cDNA synthesis kit (Thermo Fisher Scientific, Monza, Italy) according to the manufacturer’s instructions. Quantitative real time RT-PCR was performed in a reaction mixture containing SYBR Green PCR Master Mix (Bio-Rad Laboratories Srl) and 25 pmol of forward and reverse primers. Sequences of all primers are indicated in Table 1. Reactions were carried out in triplicates using a CFX96 Real Time System (Bio-Rad Laboratories Srl). Melt curve analysis was performed for each gene to ensure the specificity of amplified products. Levels of gene expression were quantified applying the 2-^ΔΔCT^ method, using GAPDH as an endogenous control, and they are expressed relative to untreated control cells.

**Table 1:** Oligonucleotide sequences used to detect the expression of reported target genes.

Target gene	Forward Primer (5′−3′)	Reverse Primer (5′−3′)
*ATG7*	ACACCAACACACTCGAGTCT	AGGGCAGGATAGCAAAACCA
*ATG5*	TGAGATAACTGAAAGGGAAGC	CATTTCAGTGGTGTGCCTTC
*ATG8*	GAAGTGGATGTTCAAGGAGG	GATGGAACCAAGTACTTCCG
*GADD45*	GAGAGCAGAAGACCGAAGG	CAGCAGGCACAACACCAC
*GAPDH*	TGCACCACCAACTGCTTAGC	GGCATGGACTGTGGTCATGAG
*IL6*	AGCCACTCACCTCTTCAGAACG	GGTTCAGGTTGTTTTCTGCCAG
*p21*	CGCTCTACATCTTCTGCCTTAGTC	AACCTCTCATTCAACCGCCTAG
*p16*	GAGCAGCATGGAGCCTTC	CATCATCATGACCTGGATCG

### Western blot analysis

Cell extracts were prepared with RIPA buffer containing proteases and phosphatase inhibitors and the concentration of cell lysates was determined using the Bradford protein assay reagent (Bio-Rad). Equal amounts of protein were resolved on SDS-polyacrylamide gel, transferred to nitrocellulose membrane (Amersham Biosciences, Milan, Italy), and then treated with the following primary antibodies: anti-Atg5 rabbit monoclonal (Cell Signaling Technology, MA, USA), anti-Atg7 rabbit monoclonal (Cell Signaling Technology, MA, USA), anti-LC3A/B rabbit monoclonal (Cell Signaling Technology, MA, USA), anti-SQSTM1/p62 rabbit monoclonal (Cell Signaling Technology, MA, USA), anti-Beclin-1 rabbit monoclonal (Cell Signaling Technology, MA, USA), anti-Phospho-S6 Ribosomal Protein rabbit monoclonal (Cell Signaling Technology, MA, USA), anti-S6 Ribosomal Protein mouse monoclonal (Cell Signaling Technology, MA, USA), anti-Phospho-AMPK rabbit monoclonal (Cell Signaling Technology, MA, USA), anti-AMPK mouse polyclonal (Cell Signaling Technology, MA, USA), anti-Cyclin D1 (Dako, Milan, Italy). Anti-Gapdh rabbit polyclonal (Santa Cruz Biotechnology) antibody was used as loading control. A secondary anti-rabbit IgG HRP-conjugated antibody and anti-mouse IgG HRP-conjugated antibody were used. Antibody complexes were detected by chemiluminescence. Imaging and densitometry analysis was performed by measuring the optical densities of specific bands using a GS-800 Calibrated Image Densitometer (Bio-Rad Laboratories Srl, Milan, Italy) or UVITEC Mini HD9 acquisition system (Alliance UVItec Ltd., Cambridge, UK).

### Protein determination by sandwich enzyme-linked immunosorbent assay

IL-6 was quantified by ELISA assay (Biotech Co., Ltd) in the supernatants of control and vitiligo melanocytes and fibroblasts according to the manufacturer’s protocol. The absolute values were extrapolated with the ELISA kit standard curve and the subsequently final results were normalized for the number of cells contained in each sample and they were expressed as picograms per 1×10^5^ cells.

### Immunofluorescence

Cells grown on coverslips were fixed with cold methanol at −20 °C and then incubated with the following primary antibodies: anti-LC3A/B rabbit monoclonal (1:100) (Cell Signaling Technology, Inc., New England Biolabs., UK), anti-Phospho-S6 Ribosomal Protein rabbit monoclonal (1:100) (Cell Signaling Technology), anti p53 mouse monoclonal (1:100) (Dako Cytomation, Glostrup, Denmark) and anti-Ki67 rabbit polyclonal (1:300) (Abcam, Cambridge Science Park, Cambridge, UK). The primary antibodies were visualized using goat anti-rabbit Alexa Fluor 555 conjugate and goat anti-mouse Alexa Fluor 488 conjugate antibodies (1:800) (Cell Signaling Technology). Coverslips were then mounted using ProLong Gold antifade reagent with DAPI (InVitrogen, Life Technologies Corporation, Oregon, USA). Fluorescence signals were analyzed by recording stained images using a CCD camera (Zeiss, Oberkochen, Germany). Quantitative analysis of LC3 positive puncta per cell was performed by counting at least 150 cells for each condition. Cell proliferation was assessed by counting Ki67-labeled cells with respect to at least 700 total cells for each condition and the result was expressed as a percentage of the control, which was set as 100. Quantitative analysis of phospho-S6 fluorescence intensity was performed using the AXIOVISION 4.7.1 software (Zeiss). For each condition, at least 7 digital images were taken from different microscopic fields and results are expressed as fluorescence intensity mean value ± SD relative to control, which was set as 1.

### Transmission electron microscopy

Cells were washed three-times in PBS and fixed with 2% glutaraldehyde in PBS for 2 h at 4 °C. Samples were postfixed with 1% osmium tetroxide in veronal acetate buffer (pH 7.4) for 1 h at 25 °C, stained with uranyl acetate (5 mg/ml) for 1 h at 25 °C, dehydrated in acetone, and embedded in Epon 812 (EMbed 812, Electron Microscopy Science, Hatfield, PA, USA). Ultrathin sections obtained with an Ultracut EMFCS ultramicrotome (Leica Microsystems, Wetzlar, Germany) were examined unstained or poststained with uranyl acetate and lead hydroxide under a Morgagni 268D transmission electron microscope (FEI, Hillsboro, OR, USA) equipped with a Mega View II charge-coupled device camera (SIS, Soft Imaging System GmbH, Munster, Germany).

### Statistical analysis

Since data followed a normal distribution, statistical differences between paired experimental groups were examined by two-tailed independent sample student’s *t*-test. Differences were considered as significant for *p ≤ 0.05 and **p ≤ 0.01. The two distributions have the same variance.

## Supplementary information

Supplementary Figure legends

Supplementary Figure 1

Supplementary Figure 2

Supplementary Figure 3
