# Supplementary material for: A protective role for autophagy in vitiligo
Source: Cell Death Dis. 2021 Mar 25;12(4):318. doi: 10.1038/s41419-021-03592-0 (PMC7994839; doi:10.1038/s41419-021-03592-0)
Supplement: Supplementary file 1 — Supplementary Figure legends [file 41419_2021_3592_MOESM1_ESM.doc]

**Supplementary Legends**

**Figure S1 Energy metabolism modulators influence autophagy.**

(A) Normalized mRNA transcripts of autophagy genes *Atg5, Atg7, Atg8* (NHMs n = 3 and VHMs n = 3) in vitiligo melanocytes treated or not with NAC (5 and 10 mM) for 24 hours; (B) Western blot of ATG5 and ATG7 with corresponding normalized densitometric values on lysates of vitiligo melanocytes after treatment with NAC (5 and 10mM) for 24 hours. A representative blot of three independent experiments is shown; (C) Immunofluorescence staining and corresponding normalized fluorescence intensity for pS6K (red) in vitiligo melanocytes before and after treatment with NAC (5mM) for 24 hours. Nuclei were counterstained with DAPI. A representative vitiligo melanocyte culture treated with NAC is shown. Scale bar: 10μm; (D) ATP assay in vitiligo melanocytes treated or not with NAC (5 and 10 mM) for 24 hours. Results are expressed as fold change relative to untreated cells value, which was set as 1 by definition (VHMs n = 3); (E) ATP assay in normal melanocytes treated or not with t-BHP (100 uM) for 24 hours. Results are expressed as fold change relative to untreated cells value, which was set as 1 by definition (NHMs n = 3). *Atg5*, autophagy related 5; *Atg7*, autophagy related 7; *Atg8*, autophagy related 8; N-acetyl-L-cystein; ATP, adenosine triphosphate; NHMs, normal human melanocytes; t-BHP, tert-butyl hydroperoxide; pS6K, Phospho S6 kinase; VHMs, vitiligo human melanocytes.

**Figure S2 Occurrence of autophagy in non lesional vitiligo fibroblasts.**

(A) Western blot analysis of LC3-II/I expression and corresponding densitometric values on vitiligo lysates normalized on control ones (NHFs n = 4 and VHFs n = 5); (B) Immunofluorescence with bright field image and corresponding normalized fluorescence intensity of pS6 kinase (red) in normal and vitiligo fibroblasts. A representative normal fibroblast culture paired with a vitiligo fibroblast one is shown. Scale bar: 10μm. LC3, (microtubule-associated protein 1) light chain 3; pS6K, Phospho S6 kinase; NHFs, normal human fibroblasts; VHFs, vitiligo human fibroblasts.

**Figure S3 Non lesional vitiligo fibroblasts responses following autophagy inhibition.**

(A) Normalized mRNA transcripts of autophagy genes Atg5, Atg7, Atg8 (NHMs n = 3 and VHMs n = 3) in vitiligo melanocyte treated or not with 3MA (5mM) at 24 and 48 hours. (B) Cell growth evaluated by MTT assay on VHMs treated with 3MA (5mM) for 24 and 48 hours. Results are expressed as fold change relative to the untreated cell value, which was set as 1 by definition (VHMs n = 3). *Atg5*, autophagy related 5; *Atg7*, autophagy related 7; *Atg8*, autophagy related 8; 3-MA, 3-methyl adenine; MTT, 3-(4,5-dimethyl-2-thiazolyl)-2,5-diphenyl-2H-tetrazolium bromide; VHMs, vitiligo human melanocytes.
