# Supplementary figures and images for: A protective role for autophagy in vitiligo
Source: Cell Death Dis. 2021 Mar 25;12(4):318. doi: 10.1038/s41419-021-03592-0 (PMC7994839; doi:10.1038/s41419-021-03592-0)

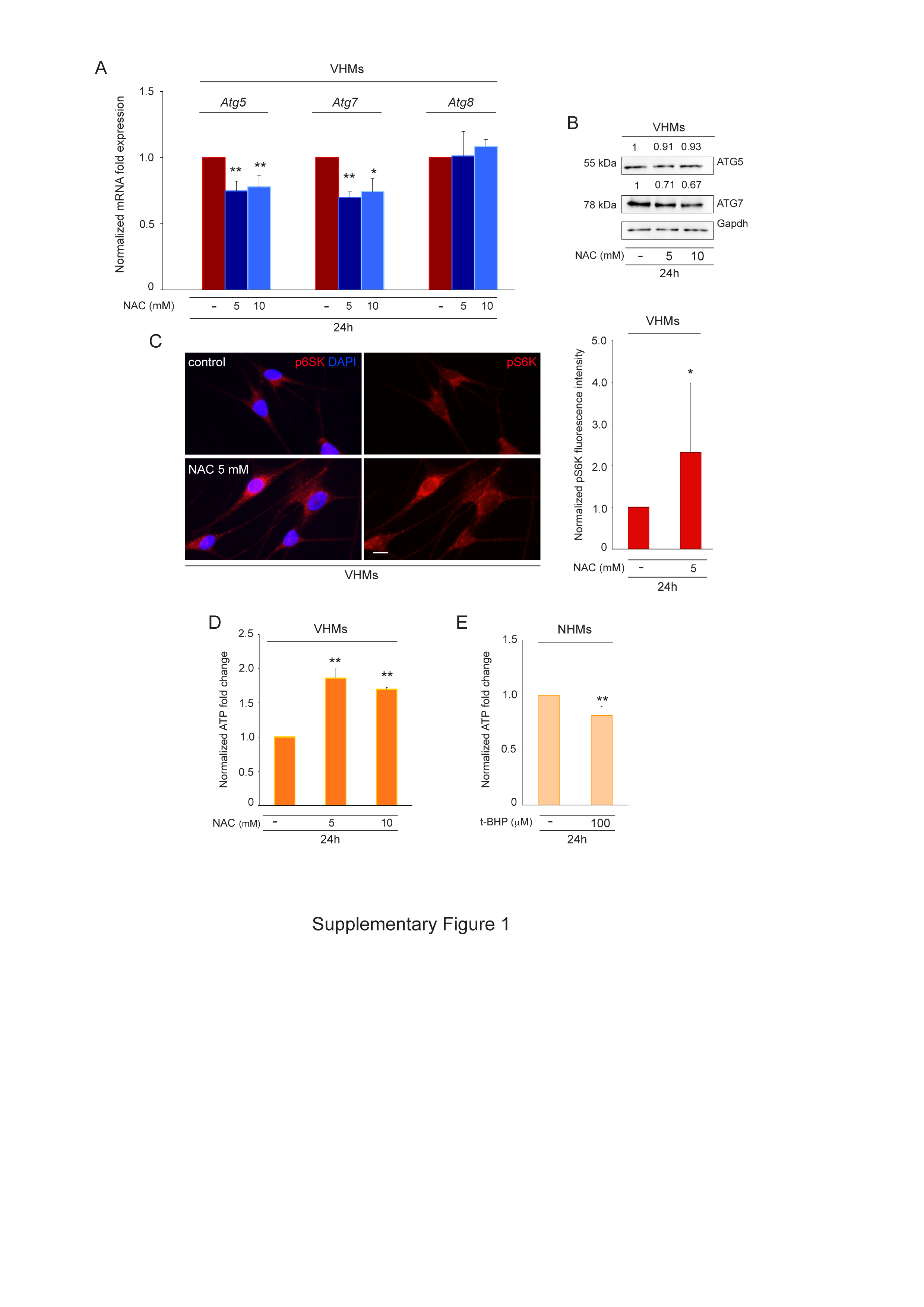

Supplement: Supplementary file 2 — Supplementary Figure 1 [file 41419_2021_3592_MOESM2_ESM.tif]

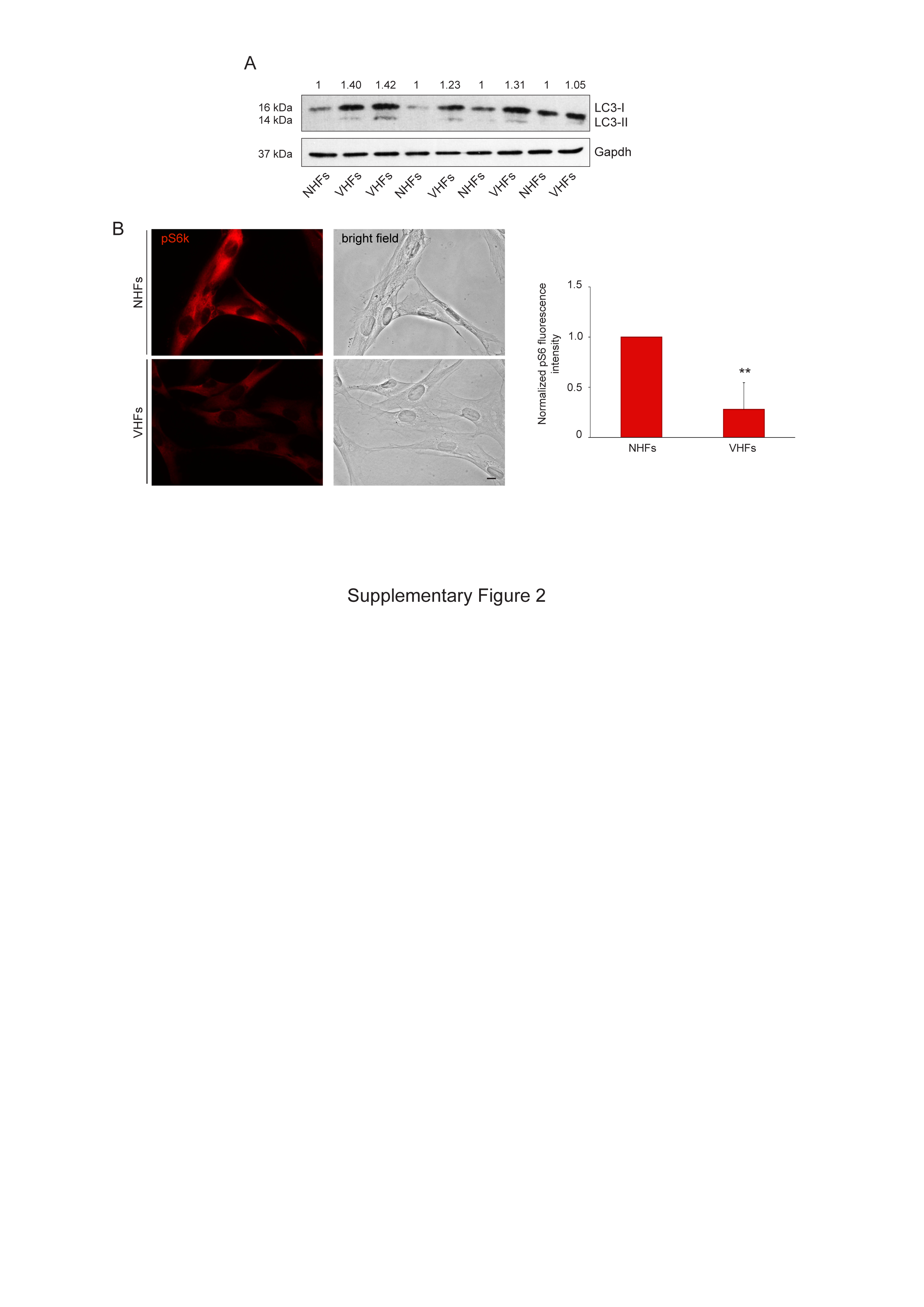

Supplement: Supplementary file 3 — Supplementary Figure 2 [file 41419_2021_3592_MOESM3_ESM.tif]

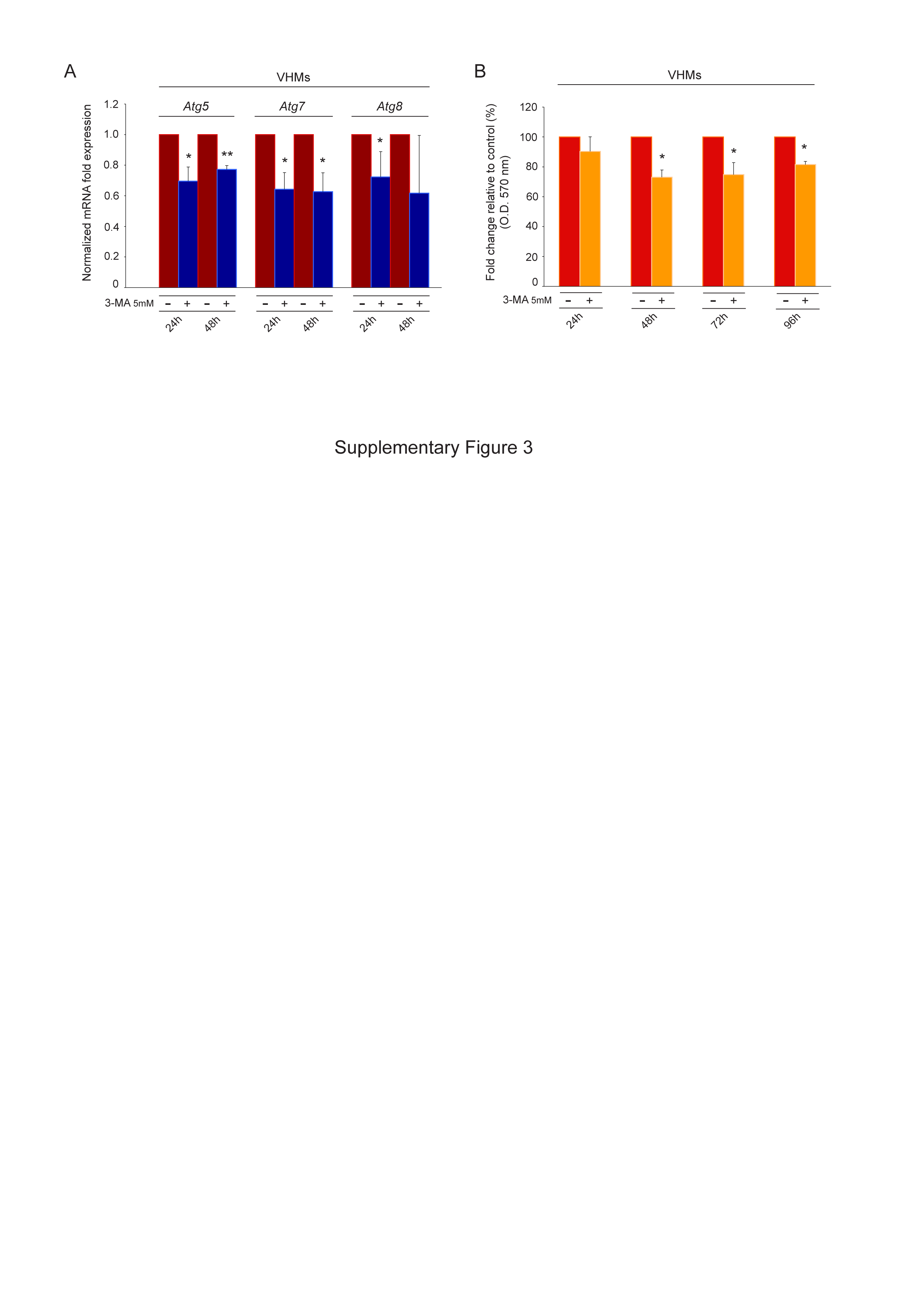

Supplement: Supplementary file 4 — Supplementary Figure 3 [file 41419_2021_3592_MOESM4_ESM.tif]
